# Epigallocatechin-3-gallate protects against osteoarthritis-induced chondrocytes dysfunction by regulating PLa2g2a

**DOI:** 10.3389/fphar.2025.1624818

**Published:** 2025-08-13

**Authors:** Mengyuan Dai, Jing Shi, Tao Wang, Sha Wan, Chen Fan, Siyu Wang, Siyuan Chen, Jiaojiao Shang, Qingquan Kong

**Affiliations:** ^1^State Key Laboratory of Biotherapy, National Clinical Research Center for Geriatrics, Center for Immunology and Hematology and General Practice Ward/International Medical Center Ward, General Practice Medical Center, West China Hospital, Sichuan University, Chengdu, China; ^2^Science and Education Section, Hospital of Chengdu Office of People’s Government of Xizang Autonomous Region (Hospital.C.X.), Chengdu, Sichuan, China; ^3^Medical College, University of Electronic Science and Technology of China, Chengdu, Sichuan, China; ^4^Biological Sample Bank, Hospital of Chengdu Office of People’s Government of Xizang Autonomous Region (Hospital.C.X.), Chengdu, China; ^5^ National Engineering Laboratory for Clean Technology of Leather Manufacture, College of Biomass Science and Engineering, Sichuan University, Chengdu, Sichuan, China; ^6^Department of Orthopedics, West China Hospital of Sichuan University, Chengdu, Sichuan, China

**Keywords:** osteoarthritis, epigallocatechin-3-gallate, transcriptome sequencing, network pharmacology, Pla2g2a

## Abstract

**Background:**

Osteoarthritis (OA) is a degenerative joint disease characterized by cartilage erosion, subchondral bone remodeling, and synovial inflammation. OA progression is driven by an imbalance between anabolic and catabolic activities in the cartilage extracellular matrix (ECM). Identifying molecular targets involved in chondrocyte (responsible for ECM homeostasis in OA) dysfunction is therefore essential for developing effective OA treatments. Notably, epigallocatechin-3-gallate (EGCG), a polyphenolic compound derived from green tea, is a known pan-assay interference compound (PAINS), which may produce non-specific *in vitro* effects (e.g., protein binding, redox interference) that challenge the interpretation of its pharmacological relevance. Thus, multi-dimensional validation (*in vitro* + *in vivo* + transcriptomic) is critical to mitigate such limitations.

**Methods:**

We investigated the effects of epigallocatechin-3-gallate (EGCG), a polyphenolic compound derived from green tea, on OA-induced dysfunction in chondrocytes. Primary chondrocytes, extracted from the knee joints of rats and constructed an OA model via IL-1β stimulation, observed cell viability and morphology upon EGCG treatment. Transcriptomic analysis was conducted to screen for differentially expressed genes. Subsequently, an OA model in rats was induced by intra-articular injection of monoiodoacetic acid (MIA), and EGCG was administered for OA treatment to validate the expression of the differentially expressed genes.

**Results:**

EGCG could reduce reactive oxygen species (ROS) levels and decreased the expression of inflammatory cytokines IL-β, MMP13, and TNF-α. Transcriptome analysis identified differentially expressed genes, and network pharmacology pinpointed Pla2g2a as a key target of EGCG. Molecular docking studies confirmed a strong binding affinity between EGCG and Pla2g2a. OA model demonstrated that EGCG treatment significantly promoted cartilage repair and increased Pla2g2a expression. *In vivo* experiments demonstrated that EGCG treatment significantly promoted cartilage repair and upregulated Pla2g2a expression, consistent with *in vitro* and transcriptomic findings—suggesting the observed effects are not solely due to PAINS interference.

**Conclusion:**

These findings underscore the therapeutic potential of EGCG in OA management via antioxidative, anti-inflammatory properties, and Pla2g2a-mediated modulation. Notably, the consistency across *in vitro*, *in vivo,* and transcriptomic data supports the biological relevance of EGCG’s effects, despite its PAINS characteristics.

## Introduction

Osteoarthritis (OA) is a degenerative joint disease characterized by the progressive erosion of articular cartilage, subchondral bone remodeling, and synovial inflammation (Jiang, 2022). As one of the most prevalent musculoskeletal disorders, OA significantly impacts the quality of life for millions of adults worldwide (Abramoff and Caldera, 2020; Global, 2022). The prevalence of OA continues to rise as populations age and obesity and injury rates increase. Although numerous studies suggest that risk factors for OA include genetics (Kiełbowski et al., 2023), mechanical stress (Zhang et al., 2022), joint injury (Collins et al., 2016), inflammation (Nedunchezhiyan et al., 2022), and metabolic abnormalities (Wei et al., 2023), the precise etiology remains elusive.

A key aspect of OA progression is the imbalance between anabolic and catabolic activities within the cartilage extracellular matrix (ECM) (Hodgkinson et al., 2021). Chondrocytes are a unique cell type in articular cartilage that maintains cartilage homeostasis primarily through dynamic regulation of extracellular matrix synthesis and degradation. However, under pathological conditions, chondrocytes undergo phenotypic changes, with abnormal metabolism being a key factor in the progression of OA. Oxidative stress is one of the main drivers of this metabolic dysfunction (Zheng et al., 2021), as it exacerbates the excessive production of reactive oxygen species (ROS), leading to mitochondrial dysfunction, alterations in signaling pathways, and changes in gene expression. This cascade promotes chondrocyte senescence and apoptosis, ultimately driving OA progression (Hu et al., 2023). Additionally, inflammatory cytokines further exacerbate chondrocyte dysfunction and cartilage degradation. Therefore, identifying key targets of abnormal chondrocyte metabolism and developing specific therapeutic agents are essential for mitigating OA progression and symptoms.

Epigallocatechin-3-gallate (EGCG), a major polyphenolic compound found in green tea, EGCG’s structure, characterized by multiple adjacent phenolic hydroxyl groups, confers such pan-assay interference compound (PAINS)-like behavior: it can non-specifically bind to proteins in cell culture media (e.g., serum albumin), interfere with redox-sensitive assays (e.g., ROS detection), or quench fluorescent signals—all of which may mask true biological effects or generate false positives (Peng et al., 2024; Bolz et al., 2021).

While EGCG has been shown to modulate various signaling pathways and molecular targets involved in inflammation and oxidative stress, the interpretability of its *in vitro* effects is inherently limited by its PAINS characteristics (Bolz et al., 2021). For example, its reported anti-inflammatory effects (e.g., inhibition of NF-κB and AP-1 signaling) could arise from non-specific protein binding rather than direct modulation of target pathways (Sah et al., 2022; Alam et al., 2022). Similarly, its antioxidant activity may reflect chemical scavenging of assay reagents rather than *bona fide* cellular ROS reduction (Liu et al., 2021). Thus, conclusions based solely on *in vitro* data risk overestimating EGCG’s pharmacological relevance.

Notwithstanding these challenges, EGCG’s therapeutic potential in OA remains compelling. Recent preclinical studies have provided compelling evidence for the therapeutic potential of intra-articular EGCG administration. Specifically, Huang et al. have demonstrated that direct intra-articular injection of EGCG significantly attenuates cartilage degeneration in a guinea pig model of spontaneous osteoarthritis, through modulation of extracellular matrix metabolism (Huang et al., 2021). Furthermore, EGCG loaded nanoparticles showed anti-inflammatory and an enhanced synovial fluid retention in *in vitro* and murine models (Yu et al., 2024).

While these pioneering studies collectively underscore the promise of localized EGCG delivery for OA management, we emphasize that their reliance on *in vitro* or single-model data may not fully address EGCG’s PAINS-related limitations. To rigorously evaluate its biological relevance, multi-dimensional validation—integrating *in vitro* mechanistic studies, *in vivo* animal models, and transcriptomic profiling—is critical.

Herein, we employ transcriptome sequencing (RNA-seq), which has emerged as a pivotal technology for elucidating key molecular targets in disease models, to investigate the role and molecular mechanisms of EGCG in improving the chondrocyte microenvironment within an OA model, aiming to provide new insights into OA alleviation and treatment. By providing a comprehensive snapshot of gene expression profiles, RNA-seq enables the identification of differentially expressed genes, alternative splicing events, and non-coding RNAs associated with various pathological conditions (Saeidian et al., 2020; Wang et al., 2009). This high-resolution approach facilitates the discovery of novel biomarkers and therapeutic targets, thereby advancing our understanding of disease mechanisms at a molecular level.

## Materials and methods

### Materials

Dulbecco’s Modified Eagle Medium: F12 (DMEM/F12), 1× phosphate-buffered saline (PBS), 100× penicillin-streptomycin, and trypsin (without EDTA) were obtained from Thermo Fisher Scientific (Waltham, MA, United States). Fetal bovine serum (FBS) was sourced from HyClone (Suzhou, China). Sprague Dawley (SD) rats were procured from Envigo Biotechnology Co., Ltd. (Chengdu, China).

### Chondrocyte isolation and culture

SD rats aged 33 days were euthanized by inhaling isoflurane after being wiped and disinfected with 75% alcohol throughout the body. Subsequently, the joint cartilage was isolated and placed in 1.5 mL sterile EP tubes, then minced using micro-scissors. 1 mL of 0.25% trypsin was added to suspend the cartilage tissue, followed by incubation at 37°C in a cell culture incubator for 30 min with agitation every 5 min. Following digestion termination, the mixture was centrifuged at 300 *g* for 10 min to remove the trypsin supernatant. Subsequently, 3 mL of 1.5% collagenase type II was added to the pellet, and the resulting cell suspension was transferred into 15 mL centrifuge tubes. The samples were then incubated in a cell culture incubator for 6–8 h, followed by a second centrifugation step at 300 × g for 10 min.Then, the resulting cell suspension was transferred to a T25 flask (passage 1) and cultured at 37°C in a cell culture incubator with 5% CO_2_. The cells were cultured in DMEM/F12 medium supplemented with 10% FBS and 1× penicillin-streptomycin. Once 90% confluency was reached, the culture was expanded at a 1:2 ratio or cryopreserve the cells. In this study, only chondrocytes within passage 3 were utilized to ensure their inherent characteristics.

### Toluidine blue staining

Passage 1 chondrocytes were digested with trypsin, centrifuged, and seeded onto a 6-well plate pre-coated with coverslips. The plate was incubated at 37°C with 5% CO_2_ in a cell culture incubator. After incubation, the cells were fixed with 95% ethanol at 4°C for 30 min, washed with 1×PBS twice, and then stained with 1% toluidine blue for 20 min. Post-staining, cells were washed with absolute ethanol, allowed to air dry, mounted with neutral resin, and observed under an inverted fluorescence microscope for subsequent photography.

### Collagen II immunohistochemistry

Similarly, cells seeded on the aforementioned 6-well plate were fixed with 4% paraformaldehyde for 20 min, followed by incubation with 3% hydrogen peroxide at room temperature for 10 min. The cells were then washed three times with 1×PBS for 5 min per wash. After incubating with normal non-immune animal serum at room temperature for 10 min, the serum was removed, and cells were incubated overnight at 4°C with 1:100 rabbit anti-collagen II primary antibody. Then, washing three times with PBS for 5 min per each, a biotinylated secondary antibody was added, and the cells were incubated at room temperature for 10 min. Following additional washes with 1×PBS three times for 5 min per each, streptavidin-biotin peroxidase was added at room temperature for 10 min. Finally, DAB staining was performed, and the cells were counterstained with hematoxylin, dehydrated, and mounted with coverslips for observation.

### Cell treatment

Cultured chondrocytes were divided into three experimental groups: (A) control group (n = 5), (B) model group (n = 5), and (C) EGCG treatment group (n = 5). To avoid the randomness of the experiment, five duplicate samples were conducted simultaneously. The model group was induced by incubating the cells with 10 ng/mL IL-1β for 24 h. For the EGCG treatment group, cells were first subjected to IL-1β induction as in the model group and subsequently treated with 10 mg/mL EGCG for an additional 24 h. Both IL-1β and EGCG were diluted in culture medium. At the conclusion of the treatment period, cells were harvested and processed for RNA sequencing and further analysis.

### ROS level detection

Chondrocytes were seeded in 6-well plates at a density of 2 × 10^5^ cells per well and cultured overnight. Subsequently, the medium was replaced, and the cells were subjected to experimental treatments. After 24 h, a 10 μM working solution of DCFH-DA prepared in serum-free medium was added and incubated with cells for 20 min at 37°C. The cells were then washed three times with serum-free medium to thoroughly remove any extracellular DCFH-DA. Finally, the intracellular ROS levels were visualized and imaged using a laser confocal microscope with an excitation wavelength of 488 nm, and the average fluorescence intensity was quantified using ImageJ software.

### IL-1β, MMP13, and TNF-α level detection

To assess the effects of EGCG on the expression of IL-1β, MMP13, and TNF-α in chondrocytes, the cell culture supernatants were collected by centrifugation at 300 *g* for 10 min to remove any cell debris and stored at −80°C for analysis. ELISA was performed on cell culture supernatants using specific ELISA kits (Ruixin biotech, Quanzhou, Fujian) according to the manufacturer’s protocols.

### Transcriptome sequencing (RNA-seq)

Cells form each group were collected and washed twice with cold 1×PBS. According to the Illumina TruSeq PE Cluster Kit protocol, total RNA was extracted, and mRNA was purified, qualifying RNA was subjected to PCR amplification for the construction of a cDNA library, and then sequenced on the Illumina NovaSeq platform to generate 150 bp paired terminal reads. Low-quality sequences and adaptor reads were removed using the Hisat2 v2.0.5. Then, according to the length of each gene and the reads count of the gene, the number of fragments sequenced per thousand base pairs (FPKM) of each gene was calculated. Gene expression was annoteted using Cufflinks software, with, default parameters applied. Gene function enrichment analysis was performed using Metscape (https://metascape.org) and KEGG function enrichment webtools.

### Target screening of EGCG

To identify key targets of epigallocatechin 3-gallate (EGCG), the PubChem database (https://pubchem.ncbi.nlm.nih.gov/) was searched using the term “epigallocatechin 3-gallate” to retrieve its SMILES molecular formula. This molecular formula was then imported into Swiss Target Prediction (http://swisstargetprediction.ch/) with the species restricted to “Rattus sapiens” to obtain predicted targets. These predicted targets were subsequently matched with differentially expressed genes identified through transcriptomic sequencing analysis. The intersection of these mapped targets was considered as the target genes for EGCG therapy in osteoarthritis. These target genes were further analyzed using gene set enrichment analysis, including Gene Ontology (GO) analysis and KEGG pathway enrichment analysis. Finally, the potential key targets were visualized and constructed into a network using Cytoscape 3.9.0.

### *In vivo* experiment

Thirty Sprague-dawley were divided into three experimental groups: (A) control group (Control group) (n = 10), (B) osteoarthritis model group (OA group) (n = 10), and (C) EGCG treatment group (EGCG group) (n = 10). Following a 7-day acclimatization period, OA group and EGCG group were received a single intra-articular injection of Monoiodoacetic acid (MIA) (2 mg/50 μL, dissolved in normal saline) to induce osteoarthritis. After 2 weeks, the EGCG group received intra-articular administration of 100 μL EGCG (10 μg/mL, dissolved in normal saline), while both the normal control (NC group) and OA group received intra-articular injections of normal saline.

One week post-treatment, the animals were anesthetized with an intraperitoneal injection of isoflurane, and blood samples were collected from the veins. Serum was separated and stored at −80°C for subsequent biochemical analysis. The knee joints were harvested and fixed in 4% paraformaldehyde for further histological examination. All experimental protocols were approved by the Ethics Committee of the Hospital of the Chengdu Office of the People’s Government of the Tibetan Autonomous Region (Approval No. KY2022-86).

### H&E staining

Histological analysis of the knee joint tissues was performed using Hematoxylin and Eosin (H&E) staining. After fixation in 4% paraformaldehyde, the knee joint tissues were decalcified in a 10% EDTA solution for 4 weeks, with the solution being refreshed weekly. Following decalcification, tissue samples were dehydrated through a series of graded ethanol solutions, cleared in xylene, and embedded in paraffin. Serial sections of 3 μm thickness were cut using a microtome and mounted on glass slides.

The tissue sections were then deparaffinized with xylene and rehydrated through a descending ethanol series. Sections were stained with hematoxylin for 5 min to highlight cell nuclei, followed by rinsing in running tap water. Slides were then dipped in acid alcohol and rinsed again before staining with eosin for 2 min to stain the cytoplasm and extracellular matrix. After thorough washing in water, the sections were dehydrated through an ascending ethanol series, cleared in xylene, and mounted with coverslips using resinous mounting medium. Histological changes in the articular cartilage and subchondral bone were observed and photographed under a panoramic slice scanner (3DHISTECH, Hungary). The severity of osteoarthritic lesions was evaluated based on established histopathological scoring systems.

### Masson staining

To assess the extent of fibrosis and collagen deposition in the knee joint tissues, Masson’s Trichrome staining was performed. After fixation and decalcification as described, serial sections of 3 μm thickness were prepared and mounted on glass slides. The sections were deparaffinized in xylene and rehydrated through descending concentrations of ethanol. The slides were then stained using Masson’s Trichrome staining kit. After staining, the sections were rinsed in distilled water, differentiated in 1% acetic acid solution for 2–5 min, dehydrated through an ascending ethanol series, cleared in xylene, and mounted with coverslips using a resinous medium. The stained sections were examined under a panoramic slice scanner (3DHISTECH, Hungary), and images were captured to evaluate the extent of collagen deposition and fibrosis in the joint tissues. The severity of osteoarthritic lesions was evaluated based on established Masson scoring systems.

### Immunohistochemistry of Pla2g2a

Immunohistochemical staining was performed to detect the expression of Pla2g2a in knee joint tissues. Following fixation, decalcification, and paraffin embedding, 5 μm tissue sections were prepared and mounted on glass slides. The sections were deparaffinized in xylene, rehydrated through a graded series of ethanol solutions, and subjected to heat-induced antigen retrieval in citrate buffer (pH 6.0) at 95°C–100°C for 20 min. To quench endogenous peroxidase activity, the sections were treated with 3% hydrogen peroxide, and non-specific binding was blocked using 5% bovine serum albumin in PBS for 30 min. Subsequently, the sections were incubated overnight at 4°C with a rabbit anti-Pla2g2a antibody (1:200, Thermo fisher, United States). The following day, the sections were incubated with a biotinylated secondary antibody (anti-rabbit IgG, Thermo fisher, United States) for 30 min at room temperature. Signal development was carried out using an avidin-biotin complex (ABC) kit followed by DAB substrate. Finally, sections were counterstained with hematoxylin, dehydrated through an ascending ethanol series, cleared in xylene, and mounted with coverslips. Stained sections were examined under a panoramic slice scanner (3DHISTECH, Hungary), and images were captured for analysis. The expression of Pla2g2a was quantified using ImageJ software by calculating the percentage of positive-stained area relative to the total tissue area.

## Statistical analysis

All data are expressed as the mean ± standard deviation. For the comparison between the groups, one-way analysis of variance (ANOVA) followed by Tukey’s *post hoc* test was used. Data were analyzed using SPSS version 21, and graphs were generated using GraphPad Prism version 8. Statistical significance was determined at p < 0.05.

## Results

### Isolation and identification of primary chondrocytes

**FIGURE 1:**
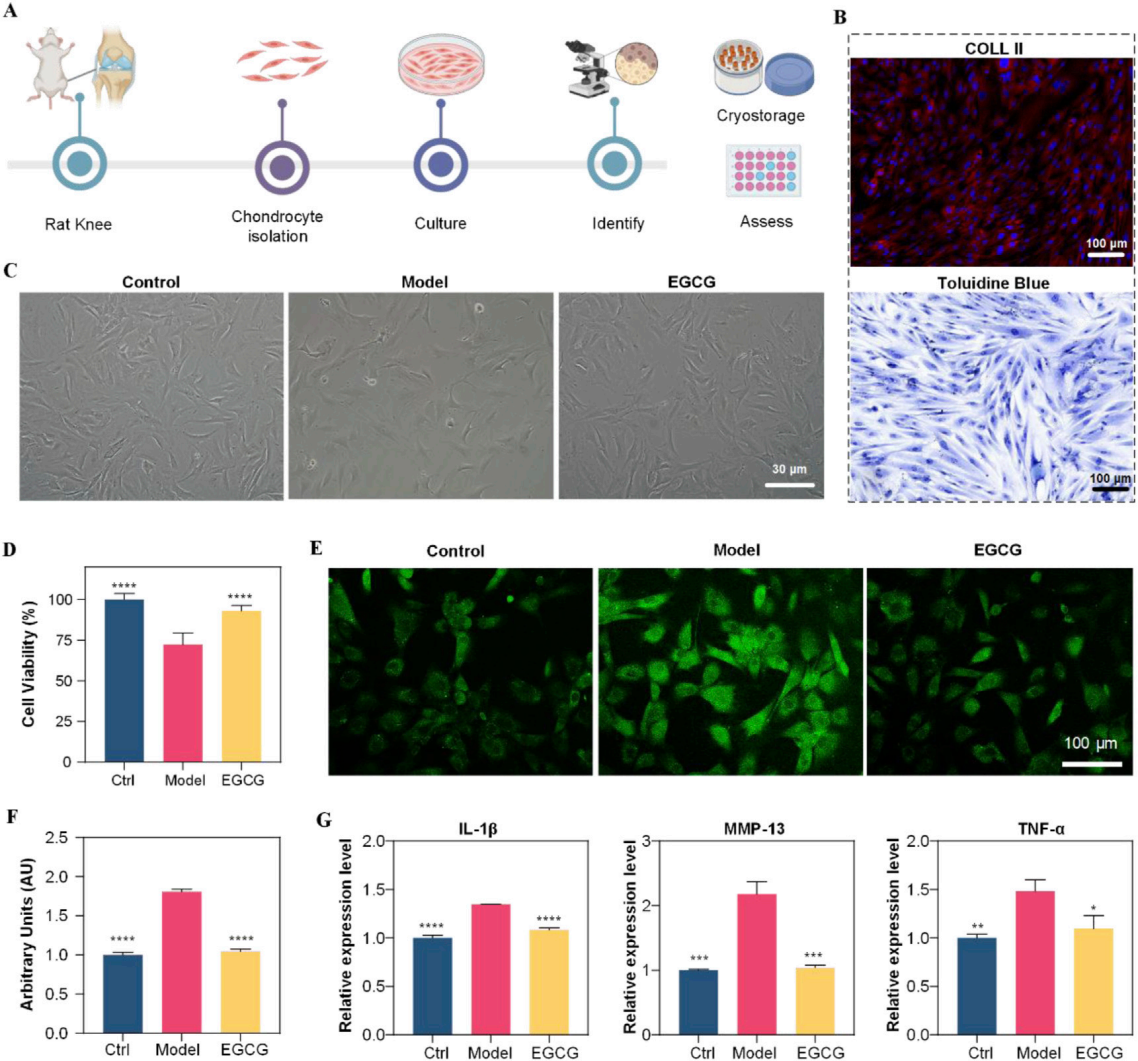
Diagram shows chondrocyte study using rats. **(A)** Workflow from rat knee to assessment. **(B)** Histology images labeled Coll II and Toluidine Blue. **(C)** Microscopic images show control, model, and EGCG-treated cells. **(D)** Bar graph of cell viability; EGCG shows moderate improvement. (E) Fluorescent images highlight chondrocyte differences. **(F)** Arbitrary units graph; EGCG shows effect. **(G)** Expression levels for IL-1β, MMP-13, TNF-α; EGCG shows decreased expression compared to model.

Primary chondrocytes were isolated from knee joints of healthy SD rats to preserve their inherent characteristics (Figure 1A). Cells were identified by collagen II immunofluorescence and toluidine blue staining. Immunofluorescence confirmed collagen II expression in the cytoplasm and membrane (Figure 1B). Toluidine blue staining revealed blue cytoplasm, blue-violet/dark blue nuclei with distinct nucleoli, and perinuclear metachromatic granules (Figure 1B). Cells displayed short spindle/long fusiform morphology with pseudopodia, typical of fibroblast-like chondrocytes. These results confirmed successful chondrocyte isolation, supporting subsequent experiments.

### Anti-inflammatory and antioxidative effects of EGCG on chondrocytes

After successful isolation and identification of primary chondrocytes, we first determined the optimal EGCG concentration by assessing its dose-dependent effects on chondrocyte viability. The data revealed that 10 μg/mL EGCG enhanced cellular viability, whereas higher concentrations (>50 μg/mL) demonstrated cytotoxicity Supplementary Figure S2; therefore, 10 μg/mL was selected for therapeutic intervention. Subsequently, to investigate EGCG’s effects on chondrocytes under osteoarthritis (OA)-mimicking conditions, cells were stimulated with IL-1β (10 ng/mL) for 24 h to establish the disease model group (Model), followed by treatment with 10 μg/mL EGCG for an additional 24 h (EGCG group). Normally cultured cells without IL-1β stimulation served as the control group (Control). As illustrated in Figure 1C, IL-1β stimulation induced significant morphological abnormalities in chondrocytes, which were partially restored upon EGCG treatment. Consistently, the cell viability of chondrocytes decreased to below 75% following IL-1β stimulation but significantly improved after EGCG treatment (Figure 1D), suggesting that EGCG exhibits reparative potential in mitigating OA-induced chondrocyte dysfunction.

Given the critical role of reactive oxygen species (ROS) in OA progression, and EGCG’s ability to scavenge intracellular ROS, we further assessed ROS levels in chondrocytes. After IL-1β stimulation, the ROS levels in chondrocytes significantly increased to approximately 1.8 times those of the control group, whereas EGCG treatment notably reduced ROS levels to near-control levels (Figures 1E,F). This finding suggests that EGCG can significantly reduce the high ROS levels induced by OA in chondrocytes.

EGCG also can inhibit the progression of inflammation by suppressing key inflammatory signaling pathways. Thus, we collected the cell culture supernatants from each group and measured the expression levels of inflammatory factors IL-1β, MMP-13, and TNF-α. As shown in Figure 1G, IL-1β stimulation significantly upregulated the expression of these inflammatory factors, while EGCG treatment notably reduced their levels. Collectively, these results demonstrate that EGCG plays a crucial role in mitigating OA-induced chondrocyte damage by reducing ROS production and suppressing inflammatory factors Expression.

### Transcriptome analysis of chondrocyte response to IL-β and EGCG treatment

**FIGURE 2:**
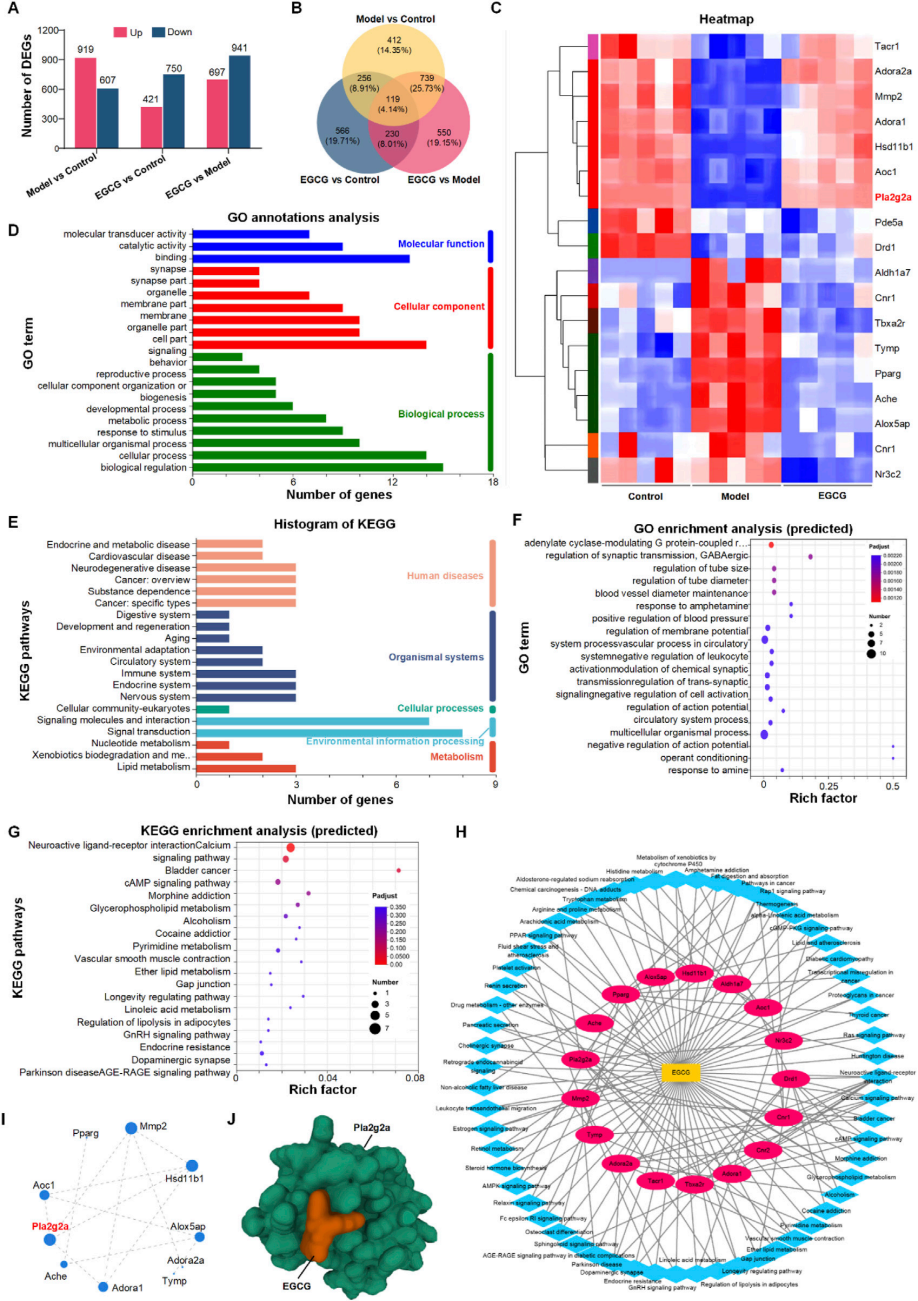
The image comprises multiple panels depicting various bioinformatics analyses. Panel **(A)** displays bar charts on differentially expressed genes in three comparisons: Model vs Control, EGCG vs Control, and EGCG vs Model. Panel **(B)** shows a Venn diagram of these comparisons. Panel **(C)** presents a heatmap highlighting gene expression patterns. Panel **(D)** features a bar chart of gene ontology annotations categorized into molecular functions, cellular components, and biological processes. Panel **(E)** includes a histogram of KEGG pathways. Panel **(F)** displays a scatter plot of predicted gene ontology enrichment. Panel **(G)** shows KEGG enrichment analysis. Panel **(H)** illustrates a network diagram of genepathway interactions, and Panel **(I)** presents a gene interaction network. Panel **(J)** is a molecular structure visualization of the gene Pla2g2a with EGCG.

Following the morphological and functional assessments of the impact of EGCG on chondrocytes under inflammatory conditions, we further explored the molecular mechanisms underlying these effects using transcriptome sequencing of mRNA from three distinct groups: Control, Model (IL-1β treated), and EGCG (EGCG-treated, 10 μg/mL). The Differentially expressed genes (DEGs) were defined using a dual-threshold criterion:|log_2_(fold change)| ≥ 1 (equivalent to 2-fold change),Adjusted p-value <0.05 (Benjamini–Hochberg FDR correction).The transcriptome analysis revealed 1526 differentially expressed genes (DEGs) between the Model and Control groups, with 919 DEGs upregulated and 607 DEGs downregulated. Comparatively, the EGCG and Control groups exhibited 1171 DEGs, including 421 upregulated and 750 downregulated genes (Figure 2A). When comparing the EGCG and Model groups, a total of 1638 DEGs were identified, of which 697 were upregulated and 941 were downregulated (Figure 2A). A Venn diagram was employed to visualize the overlap of DEGs among the three groups, highlighting 119 shared DEGs (Figure 2B).

### Identification and functional analysis of EGCG targets for alleviating IL-1β effects in chondrocytes

**FIGURE 3:**
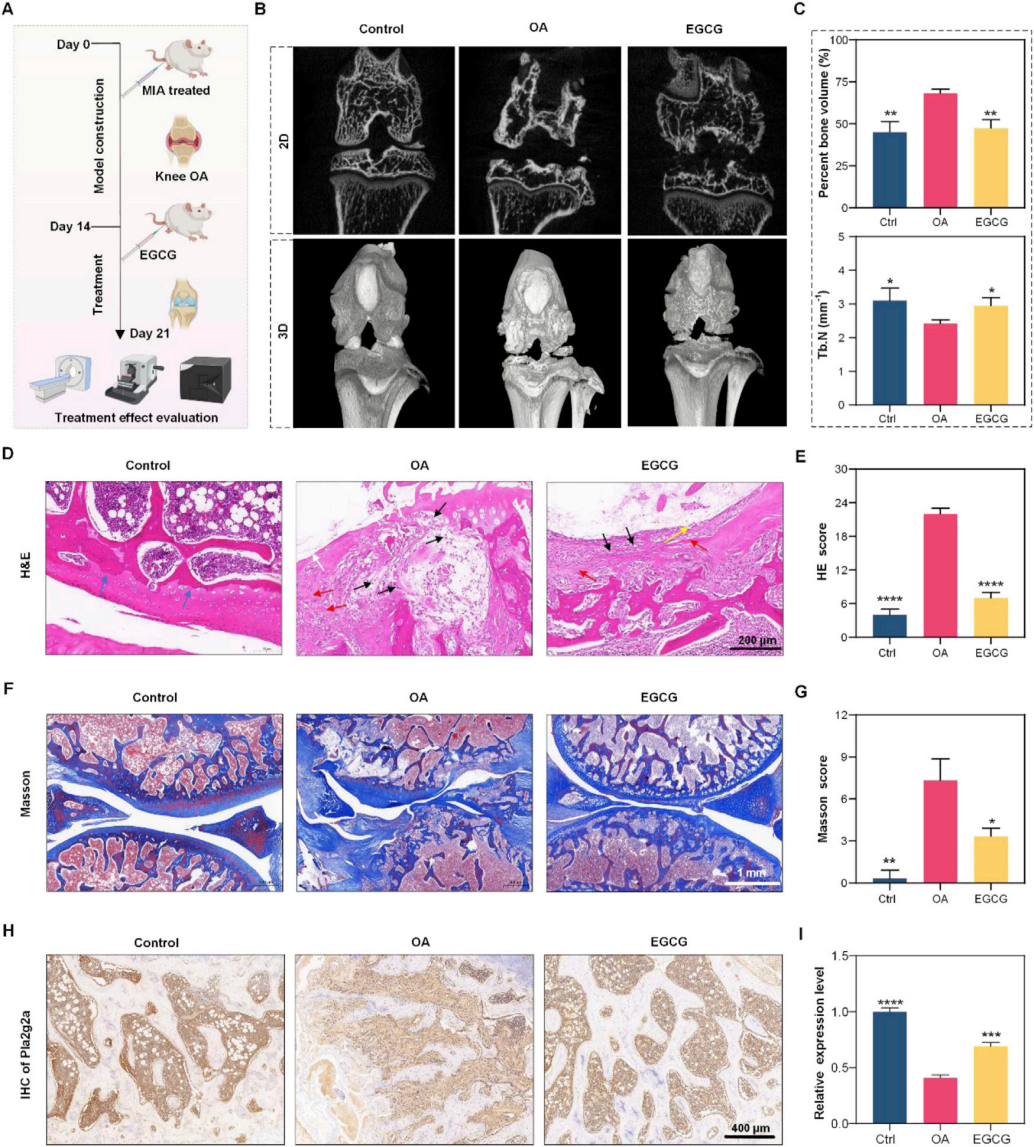
Experiment overview with multiple panels displays osteoarthritis (OA) research. Panel **(A)** describes the experimental timeline with mice undergoing different treatments, including MIA and EGCG. Panel **(B)** shows 2D and 3D images comparing bone structures in control, OA, and EGCG-treated groups. Panel **(C)** includes graphs showing bone volume and thickness data. Panel **(D)** presents H&E stained tissue images, while panel **(E)** provides H&E scores. Panel **(F)** shows Masson-stained images, and panel **(G)** includes Masson scores. Panel **(H)** displays IHC images of Pnliprp2 expression, and panel I presents its relative expression levels. *p < 0.05, **p < 0.01, ***p < 0.001, *p < 0.0001.

To expedite the identification of potential EGCG targets for mitigating the effects of IL-1β on chondrocytes, we utilized network pharmacology to identify 95 EGCG-associated targets relevant to OA (Supplementary Table S1). These targets were then correlated with the 119 differentially expressed genes identified in Figure 3B, leading to the selection of 18 potential EGCG targets for alleviating IL-1β-induced chondrocytes (Figure 2C). Detailed gene information is provided in Supplementary Table S2. Functional annotation analysis of these 18 genes revealed that Gene Ontology (GO) annotations were primarily linked to biological regulation, cellular processes, cell components, and binding (Figure 2D). Kyoto Encyclopedia of Genes and Genomes (KEGG) annotations highlighted signaling molecules and interactions, as well as signal transduction pathways (Figure 2E). Further functional enrichment analysis indicated that GO enrichment was mainly related to multicellular organismal processes (Figure 2F), while KEGG enrichment pathways focused on neuroactive ligand-receptor interactions (Figure 2G). Subsequently, KEGG pathway information was further used to construct a “component-pathway-target” network with Cytoscape 3.7.2, illustrating the complex interactions of EGCG in counteracting IL-1β effects. The network comprises 32 nodes and 138 connections (Figure 2H). By Integrating protein-protein interaction (PPI) network analysis with “target-pathway” network of KEGG, CytoNCA was utilized to calculate the “Degree” score, identifying Mmp2, Hsd11b1, and Pla2g2a as core targets with a Degree greater than 4 (Figure 2I).

### Identifying Pla2g2a as a central target of EGCG for OA therapy

To determine the key targets of EGCG in OA therapy, we used the SERING database to identify 9 central genes with a connectivity index greater than 3. These genes, including Pla2g2a, Alox5, Aldh2, Aoc1, Maob, Maoa, Htr2a, and DRD2, were markedly modulated by EGCG in the context of OA (refer to Supplementary Figure S1). A subsequent comparative analysis parallels these 9 pivotal osteoarthritis-associated genes with 3 previously identified chondrocyte-specific targets—namely, Mmp2, Hsd11b1, and Pla2g2a—revealed substantial overlap with Pla2g2a. Such convergence highlights the pivotal role of Pla2g2a, affirming it as a critical molecular target in the alleviation of chondrocyte inflammation via EGCG in OA therapy.

Additionally, we assessed the binding affinity of EGCG for its target gene, Pla2g2a, through molecular docking analysis. We utilized AutoDock Vina v.1.2.2 to calculate the binding posture and interaction dynamics between EGCG and Pla2g2a, including the calculation of binding energy. The findings revealed that EGCG interacts with Pla2g2a predominantly via stable hydrogen bonds and substantial electrostatic interactions. As shown in Figure 2J, the conformational energy of the EGCG-target protein complex was relatively low (−6.97 kcal/mol), signifying a strong binding affinity between EGCG and Pla2g2a.

### EGCG enhances cartilage repair in OA model via Pla2g2a.

Based on these findings, our preliminary investigation into the regulatory effect of EGCG on Pla2g2a expression in chondrocytes within an OA model revealed that EGCG upregulates Pla2g2a levels (Supplementary Figure S3), supporting its therapeutic potential for OA intervention. Subsequently, we further assessed the impact of EGCG on cartilage repair using an MIA-induced osteoarthritis model in the knee joints of SD rats. Initially, OA was induced in the OA group via intra-articular injection of MIA, followed by the treatment of EGCG. A control group received an equivalent volume of saline without MIA induction. Cartilage repair was evaluated on day 7 using multiple methodologies (Figure 3A). Micro-CT analysis highlighted significant bone defects post-MIA induction, which were markedly repaired after EGCG treatment (Figure 3B). Further analyses of percent bone volume and trabecular number demonstrated substantial improvements with EGCG treatment, consistent with the Micro-CT findings (Figure 3C).

Subsequent assessments focused on cartilage tissue cells and matrix damage. Hematoxylin and eosin (H&E) staining revealed disorganization and uneven cell distribution, lighter matrix staining, and reduced cell density in the OA group, with notable improvements following EGCG treatment, indicating effective cartilage repair (Figure 3D). H&E staining scores further confirmed the significant role of EGCG in promoting cartilage tissue repair (Figure 3E). Masson staining presented extensive collagen fiber structure damage in the OA group, which was substantially repaired by EGCG, restoring orderly collagen fiber alignment and clear tissue structure without abnormal deposits (Figure 3F). This was supported by Masson staining scores, underscoring the efficacy of EGCG in collagen fiber repair (Figure 3G). Additionally, analysis of Pla2g2a expression revealed a significant increase after EGCG treatment compared to its significant reduction in OA group (Figures 3H,I), indicating the therapeutic potential of EGCG in OA treatment through Pla2g2a mediation in cartilage cells.

## Discussion

Osteoarthritis (OA) is a prevalent degenerative joint disorder characterized by articular cartilage degeneration and subchondral bone changes (Yao et al., 2023; Abramoff and Caldera, 2020; Hu et al., 2019). EGCG, a key polyphenol in green tea, has been recognized for its anti-inflammatory and antioxidant properties in inflammatory diseases (Ansari et al., 2020; Xie et al., 2020; Prasanth et al., 2019).

This study investigates the anti-inflammatory effects of EGCG on osteoarthritis, explores its underlying molecular mechanisms, and seeks therapeutic targets to provide a basis for early clinical diagnosis, treatment, and prevention.

However, it is critical to contextualize these *in vitro* observations within the framework of EGCG’s well-documented pan-assay interference compound (PAINS) characteristics. PAINS, such as EGCG, often produce non-specific “positive” results in cell-based assays due to their physicochemical properties—including non-specific protein binding, redox interference, or fluorescence quenching. For instance, EGCG’s multiple phenolic hydroxyl groups may bind to serum proteins in cell culture media, reducing its bioavailability and potentially masking true target interactions; alternatively, its redox activity could directly scavenge ROS detection reagents (e.g., DCFH-DA), leading to false reductions in measured ROS levels.

To address these limitations, our study employed a multi-dimensional validation strategy to distinguish EGCG’s *bona fide* biological effects from PAINS-induced artifacts.The main approach was to jointly study the anti-inflammatory effect of EGCG on osteoarthritis *in vitro* and *in vivo*, and then combine high-throughput transcriptomic analysis to explore its molecular mechanism and identify therapeutic targets. To provide a basis for early clinical diagnosis, treatment and prevention.

Our research has successfully highlighted the substantial protective effects of EGCG, a predominant polyphenolic compound in green tea, against OA-induced chondrocyte dysfunction. By isolating primary chondrocytes and constructing an OA model through IL-1β stimulation, we demonstrated that EGCG treatment significantly restores chondrocyte viability and morphology. EGCG also markedly reduces ROS levels and diminishes the expression of inflammatory cytokines, showcasing its strong antioxidative and anti-inflammatory properties. These findings indicate the ability of EGCG to counteract the cellular and molecular mechanisms underlying OA pathogenesis. Further transcriptome sequencing revealed that EGCG modulates numerous genes related to inflammation and oxidative stress, illustrating its extensive impact on cellular pathways pertinent to OA. Network pharmacology analysis pinpointed Pla2g2a as a key target of EGCG in reducing the chondrocytes damage in the OA model. The confirmed strong binding affinity between EGCG and Pla2g2a, as confirmed by molecular docking analysis, emphasizes the therapeutic potential of EGCG in treating OA, suggesting a specific molecular mechanism behind its protective effects and offering a targeted approach for therapeutic intervention.

EGCG, the primary bioactive polyphenol in green tea, exerts multi-target modulation of inflammatory and oxidative stress pathways. Building on this foundation, our integrated transcriptomic-network pharmacology approach reveals a novel mechanistic link between EGCG and Pla2g2a, a pivotal upstream regulator of the arachidonic acid metabolic pathway. Unlike the well-characterized EGCG-mediated inhibition of NF-κB or activation of Nrf2, our findings uniquely demonstrate that EGCG attenuates lipid mediator synthesis via Pla2g2a targeting, thereby addressing a critical gap in understanding polyphenol-mediated lipid metabolic regulation in OA.

Recent advances (Ali et al., 2023) have highlighted polyphenols’ dual roles in lipid metabolism modulation (e.g., hydroxytyrosol suppressing PI3K/Akt/mTOR) and epigenetic remodeling (e.g., olive phenols regulating CB1 gene methylation). Our discovery that EGCG specifically governs arachidonic acid metabolism through Pla2g2a provides a complementary mechanistic layer, robustly supporting the emerging ‘multi-dimensional regulation’ theory of polyphenols. This paradigm shift underscores EGCG’s capacity to synergistically target inflammatory cascades at both transcriptional and metabolic levels, offering a novel therapeutic dimension for OA management.

Despite EGCG’s established antioxidant/anti-inflammatory properties, our study innovates with a de-redundant screening strategy (topology-based network filtering and machine learning-driven target prediction), enabling precise identification of context-specific targets often missed by traditional methods. Integrating RNA-seq and network pharmacology, we bypassed canonical pathways to identify the covert node PLA2G2A, confirming EGCG targets this protein via natural product pathways—offering a novel perspective for low-toxicity OA precision therapies. Our multi-dimensional design (*in vitro* + *in vivo* + transcriptomic) overcomes prior limitations (e.g., sole *in vitro* data and PAINS neglect), strengthening evidence for EGCG’s relevance despite its PAINS properties. Residual uncertainties (e.g., off-target effects) persist, and clinical validation (pharmacokinetics, dose-response) is needed to confirm translational potential.

In summary, our findings establish EGCG as a promising therapeutic agent for OA by demonstrating its PLA2G2A regulation, thereby alleviating disease symptoms. This study not only validates EGCG’s potential as a precision treatment option but also provides a novel framework for identifying context-specific therapeutic targets through integrated de-redundant screening (topology-based filtering + machine learning prediction) and multi-dimensional validation (*in vitro* + *in vivo* + transcriptomic). Future investigations should prioritize clinical trials to evaluate EGCG’s efficacy, safety, and pharmacokinetic profiles in OA patients, while exploring synergistic effects with existing therapies to optimize treatment outcomes.

## Data Availability

The data presented in the study are deposited in the Genome Sequence Archive (Genomics, Proteomics & Bioinformatics 2021) in National Genomics Data Center (Nucleic Acids Res 2022), China National Center for Bioinformation/Beijing Institute of Genomics, Chinese Academy of Sciences (GSA: CRA011921) that are publicly accessible at https:// ngdc.cncb.ac.cn/gsa.
