# Supplementary material for: Epigallocatechin-3-gallate protects against osteoarthritis-induced chondrocytes dysfunction by regulating PLa2g2a
Source: Front Pharmacol. 2025 Aug 13;16:1624818. doi: 10.3389/fphar.2025.1624818 (PMC12380777; doi:10.3389/fphar.2025.1624818)
Supplement: Supplementary file 1 [file DataSheet1.pdf]

Supplymentary Information

Epigallocatechin-3-gallate protects against osteoarthritis-induced chondrocytes dysfunction by regulating PLa2g2a

Mengyuan Dai[1†], Jing Shi[2†], Jinghong Xian[3†], Tao Wang[4], Sha Wan[2], Chen Fan[2], Siyu Wang[5], Siyuan Chen[5], Jiaojiao Shang[6*], Qingquan Kong[2, 7*]

[1] National Clinical Research Center for Geriatrics, Center for Immunology and Hematology and General Practice Ward/International Medical Center Ward, General Practice Medical Center, State Key Laboratory of Biotherapy, West China Hospital, Sichuan University, Chengdu, China.

[2] Science and Education Section, Hospital of Chengdu Office of People's Government of Xizang Autonomous Region (Hospital.C.X.), Chengdu, Sichuan, China.

[3] Department of Pulmonary and Critical Care Medicine, West China Hospital, State Key Laboratory of Respiratory Health and Multimorbidity, Sichuan University, Chengdu, Sichuan, China[4] Medical college, University of Electronic Science and Technology of China, Chengdu, Sichuan, China.

[5] Biological sample bank, Hospital of Chengdu Office of People's Government of Xizang Autonomous Region (Hospital.C.X.), Chengdu 610041, China;

[6] National Engineering Laboratory for Clean Technology of Leather Manufacture, College of Biomass Science and Engineering, Sichuan University, Chengdu, Sichuan, China.

[7] Department of Orthopedics, West China Hospital of Sichuan University, Chengdu, Sichuan, China.

[†] Mengyuan Dai, Jing Shi and Jinghong Xian contributed equally to this work.

Corresponding author Email: kongspine@126.com; jiaoj.shang@scu.edu.cn

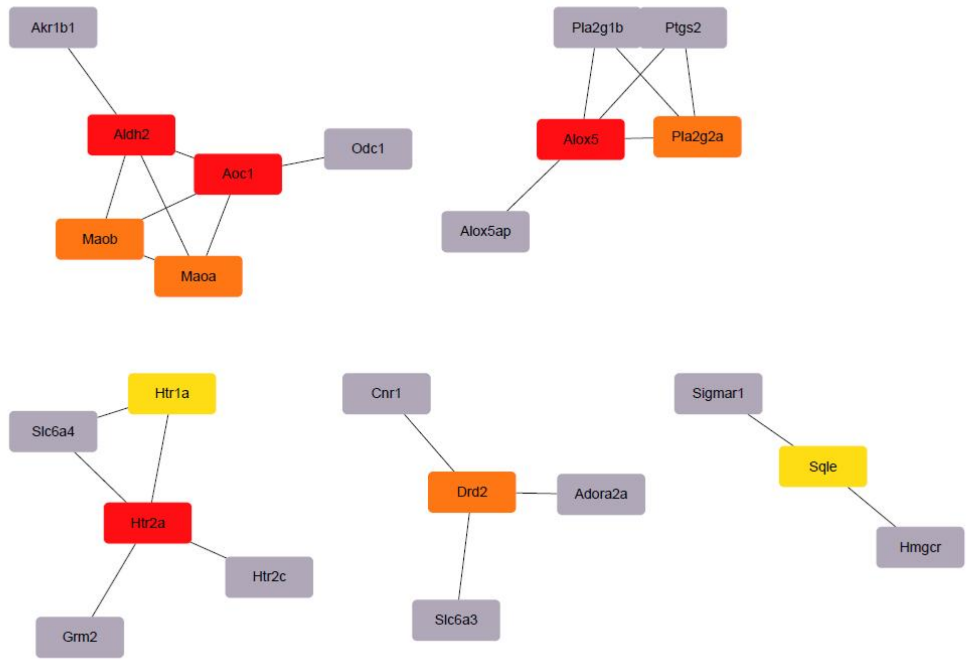

Fig. S1. Network Diagram of Key EGCG Targets in OA Therapy Identified via SERING Database. This network depicts genes modulated by EGCG in osteoarthritis (OA). Central genes (connectivity index >3, marked in bold) include Pla2g2a, Alox5, Aldh2, Aoc1, Maob, Maoa, Htr2a, and DRD2. Nodes represent genes; edges represent interactions. Node size and color distinguish connectivity levels and functional groups, respectively. Interconnected genes (e.g., Akr1b1, Odc1, Alox5ap) further illustrate EGCG's regulatory network in OA.

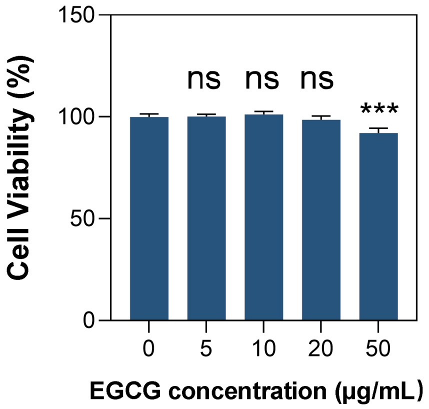

Fig. S2. Cell Viability After Treatment with Varying Concentrations of EGCG. Bar graph illustrating the percentage of chondrocyte viability after exposure to EGCG concentrations of 0, 5, 10, 20, and 50 μg/mL. Statistical significance markers: "ns" indicates no significant difference; "***" denotes extremely significant difference ($p < 0.001$).

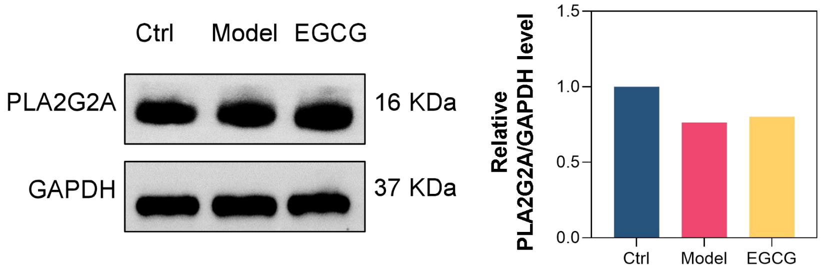

Fig S3. Western-Blot analysis of Pla2g2a protein expression. Representative Western blot bands of Pla2g2a (16 kDa) and loading control GAPDH (37 kDa) across experimental groups (Left). And bar graph quantifying relative Pla2g2a/GAPDH levels (Right).
